# Supplementary material for: 3D molecular generative framework for interaction-guided drug design
Source: Nat Commun. 2024 Mar 27;15:2688. doi: 10.1038/s41467-024-47011-2 (PMC10973397; doi:10.1038/s41467-024-47011-2)
Supplement: Supplementary file 1 — Supplementary Information [file 41467_2024_47011_MOESM1_ESM.pdf]

Electronic Supplementary Information for:
3D Molecular Generative Framework for Interaction-guided Drug Design

Wonho Zhung[1], Hyeongwoo Kim[1], Woo Youn Kim[1,2,3*]

[1]Department of Chemistry, KAIST, 291, Daehak-ro, Yuseong-gu, Daejeon, 34141, Republic of Korea,

[2]AI Institute, KAIST, 291, Daehak-ro, Yuseong-gu, Daejeon, 34141, Republic of Korea,

[3]HITS Inc., 124, Teheran-ro, Gangnam-gu, Seoul, 06234, Republic of Korea,

[*]Corresponding author; E-mail: wooyoun@kaist.ac.kr.

Supplementary Methods

1. Model architectures of DeepICL

(a) Node and edge features

The input node features of ligand and protein atoms, $\mathbf{X}_i^l \in \mathbb{R}^{F^l}$ and $\mathbf{X}_j^p \in \mathbb{R}^{F^p}$, are used as described in the Supplementary Table 1. Each of the features is represented as a one-hot vector of a corresponding category. Only an atom type is used for a ligand atom feature, while more informative features are used for a protein atom feature by concatenating every one-hot vector. The resulting feature dimensions of a ligand and a protein atom are $F^l = 9$ and $F^p = 51$, respectively. Initial node features of a ligand and a protein are then embedded into a hidden dimension F^h with a single linear layer.

Ligand atom feature, $\mathbf{X}_i^l$	Available list
Atom type	C, N, O, F, P, S, Cl, Br, stop (one-hot)
Protein atom feature, $\mathbf{X}_j^p$	Available list
Atom type	C, N, O, F, P, S, Cl, Br, *else* (one-hot)
Atom degree	0, 1, 2, 3, 4, *else* (one-hot)
Hybridization	s, sp, sp^2, sp^3, sp^3d, sp^3d^2, *else* (one-hot)
Formal charge	-2, -1, 0, 1, 2, 3, *else* (one-hot)
Amino acid type	G, A, V, L, I, C, M, F, Y, W, P, S, T, Q, N, D, E, H, R, K, *else* (one-hot)
Aromaticity	0 or 1

Supplementary Table 1. The used node features of ligand and protein atoms with their available item lists to construct a one-hot vector.

We used the Gaussian expansion of a distance between the i-th and j-th nodes as an edge feature, $\mathbf{e}_{ij} := \{e_n(d_{ij})\}_{n=1}^N$. Each Gaussian distribution is located at each center value of a distance bin, where a total distance is divided into N bins with a spacing of $\Delta\mu$. The smoothness of the expansion is controlled by γ. Formally,

$$e_n(d_{ij}) = \frac{\mathrm{e}^{-\gamma(d_{ij}-n\Delta\mu)^2}}{\sum_{n'=0}^{N-1} \mathrm{e}^{-\gamma(d_{ij}-n'\Delta\mu)^2}}. \tag{1}$$

Specific values of each hyper-parameter are summarized in the Supplementary Table 2.

(b) $\boldsymbol{E}(3)$-Invariant interaction network architecture

While updating the node features of a protein and a ligand, we devise an $E(3)$-invariant interaction network that can propagate the inter- and intra-molecular messages between a protein and a ligand. A single layer of the network consists of three steps: inter- and intra-molecular message calculation and aggregation (eqs. (2) to (5)), gate coefficient calculation (eq. (6)), and node feature update (eq. (7)). Formally,

$$\mathbf{m}_{k\to i} = \phi_{\text{intra}}(\mathbf{h}_k^{\text{l}},\ \mathbf{h}_i^{\text{l}},\ \mathbf{e}_{ki}),\ \mathbf{m}_{l\to j} = \phi_{\text{intra}}(\mathbf{h}_l^{\text{p}},\ \mathbf{h}_j^{\text{p}},\ \mathbf{e}_{lj}), \tag{2}$$

$$\boldsymbol{\mu}_{l\to i} = \phi_{\text{inter}}(\mathbf{h}_l^{\text{p}},\ \mathbf{h}_i^{\text{l}},\ \mathbf{e}_{li}),\ \boldsymbol{\mu}_{k\to j} = \phi_{\text{inter}}(\mathbf{h}_k^{\text{l}},\ \mathbf{h}_j^{\text{p}},\ \mathbf{e}_{kj}), \tag{3}$$

$$\mathbf{m}_i = \sum_k \mathbf{m}_{k\to i},\ \mathbf{m}_j = \sum_l \mathbf{m}_{l\to j}, \tag{4}$$

$$\boldsymbol{\mu}_i = \sum_l \boldsymbol{\mu}_{l\to i},\ \boldsymbol{\mu}_j = \sum_k \boldsymbol{\mu}_{k\to j}, \tag{5}$$

$$z_i = \psi_{\text{gate}}(\mathbf{m}_i,\ \boldsymbol{\mu}_i),\ z_j = \psi_{\text{gate}}(\mathbf{m}_j,\ \boldsymbol{\mu}_j), \tag{6}$$

$$\mathbf{h}_i^{\text{l}\prime} = \chi^{\text{l}}\big(\mathbf{h}_i^{\text{l}},\ z_i \cdot \mathbf{m}_i + (1-z_i)\cdot \boldsymbol{\mu}_i\big),\ \mathbf{h}_j^{\text{p}\prime} = \chi^{\text{p}}\big(\mathbf{h}_j^{\text{p}},\ z_j \cdot \mathbf{m}_j + (1-z_j)\cdot \boldsymbol{\mu}_j\big). \tag{7}$$

For convenience, subscripts i, k denote ligand atom indices, and j, l denote protein atom indices. ϕ_{intra}, ϕ_{inter}, and ψ_{gate} are learnable models shared on both a ligand and a protein. ϕ_{intra} and ϕ_{inter} are multi-layer

perceptrons (MLPs) activated by sigmoid linear units (SiLUs)[1], while ψ_{gate} is a single linear layer followed by a sigmoid function. Intramolecular message $\mathbf{m}$ and intermolecular message $\boldsymbol{\mu}$ are linearly interpolated by gate coefficients z_i and z_j and then used to update the current node hidden states. The hidden vectors for a ligand and a protein, $\mathbf{h}^{\text{l}}$ and $\mathbf{h}^{\text{p}}$, are updated by χ^{l} and χ^{p} of gated recurrent units (GRUs)[2], yielding the updated hidden vectors $\mathbf{h}^{\text{l}'}$ and $\mathbf{h}^{\text{p}'}$, respectively.

(c) Atom type and position prediction model

After node feature updates through multiple layers of $E(3)$-invariant interaction networks, the features are joined with a latent vector, z. After that, only protein node features are additionally joined with an interaction condition, $\mathbf{I}$. The resulting features then undergo fully connected layers into dimensions of type and distance distributions individually. We used SiLU as an activation function, where final prediction outputs go through a softmax function for normalization.

2. Training details

(a) Loss scheduling

A decoder that generates an output in an autoregressive fashion can be susceptible to the KL-vanishing problem, which might cause undesired model behaviors such as mode collapse[3, 4]. To mitigate this KL-vanishing problem, one can employ an annealing schedule for the KL divergence term. Various strategies for the annealing schedules have been proposed[3–6]. In our study, we adopted the simplest monotonic KL annealing, gradually increasing the weight of the KL divergence term up to a predefined value during training. Formally, the weight $\beta(t)$ is scheduled at the t-th epoch as:

$$\beta(t) = \beta_f + (\beta_i - \beta_f) \cdot \eta^t, \tag{8}$$

where β_i is the initial weight, β_f is the final wight, and η is the weight annealing factor. Their specific values used in this work can be found in the Supplementary Table 2. Thus, our final loss function can be written as follows:

$$\ell_{total}(t) = \ell_{recon} + \beta(t) \cdot \ell_{reg}. \tag{9}$$

In addition, we used `ReduceLROnPlateau` module implemented in PyTorch[7], which reduces a learning rate if there is no improvement in the validation loss for a fixed number of training epochs until the learning rate reaches a minimum threshold value. Specific hyper-parameters are also summarized in Supplementary Table 2.

3. Sampling details

(a) Controlling the randomness

In conditional deep generative models, ensuring diversity and novelty in the sampled outputs is an important concern. One simple way to increase them is by introducing some randomness into the sampling process. In our study, we control the randomness of the proposed ligands by employing a temperature factor and a roto-translational noise to the sampling process using DeepICL. Although the randomness of the sampling process affects the validity, our model produces approximately 99% of chemically valid ligands from the provided experimental settings in Supplementary Table 2.

i. Temperature factor As in the work of G-SchNet[8], we use an additional temperature factor that allows for controlling the randomness of the generation. We reformulate eqs. (11) and (12) in the main text (Section 4.2.5), which is to define the likelihood of the next atom type and its position in the sampling process, to introduce temperature factors τ_{type} and τ_{pos}:

$$\tilde{p}(\mathbf{X}_t|C_{t-1}, \mathbf{I}, z) = \frac{1}{a}\exp\left(\frac{\log p(\mathbf{X}_t|C_{t-1}, \mathbf{I}, z)}{\tau_{type}}\right), \tag{10}$$

$$\tilde{p}(\mathbf{r}_t|\mathbf{X}_t, C_{t-1}, \mathbf{I}, z) = \frac{1}{b}\exp\left(\frac{\log p(\mathbf{r}_t|\mathbf{X}_t, C_{t-1}, \mathbf{I}, z)}{\tau_{pos}}\right), \tag{11}$$

where a and b are normalization constants. Increasing τ_{type} and τ_{pos} will smoothen the predicted probability distributions, adding more randomness to the next atom prediction, whereas small values will produce sharper distributions, leading to less randomness.

ii. Adding roto-translational noise We adopt an additional method that introduces a random noise to the initial state of the sampling process. We use a different random noise depending on the ligand design task being considered; in the case of *de novo* ligand design, a translational noise is added to the given center-of-mass, whereas a roto-translational noise is added to the initial ligand core structure for a ligand elaboration task. A translational noise is simply a vector sampled from a Gaussian distribution in 3D space centered at the origin with variance σ_t, which is a hyper-parameter to control the randomness of the translational noise. Then, the center-of-mass is moved by the vector.

For applying extra rotational noise, the rotation axis is sampled from a uniform distribution, and its rotational angle is sampled from a Gaussian distribution centered at the origin with variance σ_r, which is a hyper-parameter to control the randomness of the rotational noise. By increasing σ_t and σ_r, the initial state will be scattered with a greater variance, appending more randomness to the ligand sampling process. The specific value of each hyper-parameter used in the experiments is summarized in the Supplementary Table 2.

(b) Determining the pocket coefficient

We introduce a pocket coefficient λ in eqs. (11) and (12) in the main text (Section 4.2.5), which controls the weight of a pocket-side prediction during the atom sampling. When the space where the next atom will be added is located far from the pocket, the dependence of ligand atom types and positions on the pocket diminishes. To reflect this, we allow the distance between a ligand and a pocket to determine the pocket coefficient. We average the distances between the ligand atom-of-interest and its k-nearest pocket atoms as described in eq. (12). Then, the pocket coefficient λ is determined by a function defined in eq. (13). The function decays as the average distance, $\bar{d}$, increases in the region where $\bar{d}$ is larger than 2.5 Å. We set its decaying coefficient so that $\lambda \simeq 1$ at $\bar{d} = 5.0$ Å.

$$\bar{d}(t^*) = \frac{1}{k} \sum_{j \in \mathcal{N}_k(t^*)} d_{t^*,j}, \tag{12}$$

$$\lambda = \begin{cases} 10 & \text{if } 0 \leq \bar{d} \leq 2.5 \text{ Å}, \\ 10 \cdot e^{-0.91(\bar{d}-2.5)} & \text{otherwise.} \end{cases} \tag{13}$$

Hyper-parameters	Descriptions	Values
k	number of nearest neighbors	8
F^h	hidden dimension	128
n_interaction_layers	number of interaction layers	6
n_fc_layers	number of FC layers	3
N	number of bins for Gaussian expansion	25, 300
$\Delta\mu$	spacing between distance bins	0.4 Å, 0.05 Å
γ	smoothness of Gaussian expansion	10, 50
τ_{type}, τ_{pos}	temperature factor	0.1, 0.1
σ_t, σ_r	variance of roto-translational noise	0.2 Å, 2°
β_i, β_f	KL-divergence loss initial and final weights	0.0, 1.0
η	KL-divergence loss annealing factor	0.2
lr	initial learning rate	10^{-3}
lr_min	minimum learning rate	10^{-6}
lr_decay	lr decaying factor	0.8
lr_tol	lr decay tolerance epochs	4

Supplementary Table 2. Descriptions of the hyper-parameters and their values used in this work.

4. Reference-free interaction conditions

We used specific criteria to designate protein atoms' interaction types in the case when no reference ligand information is available. Atoms involved in salt bridge anions and cations, hydrogen bond acceptors, and donors are selected by using SMARTS descriptors summarized in the Supplementary Table 3. Since only particular motifs that appear on amino acid chains are known to have cations or anions, we set SMARTS patterns to fully cover them, even their resonance structures. Halogen atoms or carbons, which are surrounded only by carbon or hydrogen atoms, are classified as hydrophobic atoms, and atoms within aromatic rings are classified as aromatic atoms.

Types	SMARTS descriptors
Anion	[O;$([OH0-,OH][CX3](=[OX1])),$([OX1]=[CX3]([OH0-,OH]))]
Cation (Lysine)	[N;$([NX3H2,NX4H3+;!$(NC=[!#6]);!$(NC#[!#6])][#6])]
Cation (Arginine)	[#7;$([NH2X3][CH0X3](=[NH2X3+,NHX2+0])<br>[NHX3]),$([NH2X3+,NHX2+0]=[CH0X3]([NH2X3])[NHX3])]
Cation (Histidine)	[#7;$([$([#7X3H+,#7X2H0+0]:[#6X3H]:[#7X3H]),<br>$([#7X3H])]1:[#6X3H]:[$([#7X3H+,#7X2H0+0]:<br>[#6X3H]:[#7X3H]),$([#7X3H])]:[#6X3H]:[#6X3]1),<br>$([$([#7X3H+,#7X2H0+0]:[#6X3H]:[#7X3H]),<br>$([#7X3H])]1:[#6X3H]:[$([#7X3H+,#7X2H0+0]:<br>[#6X3H]:[#7X3H]),$([#7X3H])]:[#6X3]:<br>[#6X3H]1)]
Hydrogen bond acceptor	[$([!#6;+0]);!$([F,Cl,Br,I]);<br>!$([o,s,nX3]);!$([Nv5,Pv5,Sv4,Sv6])]
Hydrogen bond donor	[!#6;!H0]

Supplementary Table 3. SMARTS descriptors for anion, cation, hydrogen bond acceptor, and donor.

5. Molecular dynamics simulation details

During the MD simulations done in this work, topology and parameter files for the ligands were generated using the GAFF-2.11 force field[9] via the OpenMM Toolkit[10]. Protein-ligand structures were solvated in a cubic box using TIP3P water models[11], extending 10 Å from the protein to provide padding. The systems were neutralized by adding Na^+ and Cl^- ions. Periodic boundary conditions were applied to the systems in the NPT ensemble using the Langevin thermostat[12]. To simulate the interactions, we employed the Amber FF14SB force field[13]. Equilibration and production runs were performed using the OpenMM toolkit. Initially, the systems underwent energy minimization followed by 1 ns of equilibration. Production runs were conducted at 303.5 K and 1 bar, using a 2 fs integration time step. Each protein-ligand complex underwent 10 ns production runs, initiated with the same initial structure but differently randomized initial velocities. To assess the stability of ligand binding to the target protein, we aligned protein backbone structures from each frame based on their heavy atom coordinates using MDtraj software[14] to capture the ligand movement only and calculate the ligand RMSDs. For each protein-ligand complex, the ligand RMSD was averaged over the entire 10 ns simulation time, yielding the averaged ligand RMSD value and its variance.

Supplementary Discussion

6. Additional discussions and examples of an interaction-aware conditioned ligand elaboration

In Section 2.2 of the main text, we only discussed the first case in which the target was BMP1; we here continue the comprehensive study of the other two examples. The second example is an elaboration of 2-(oxan-3-yloxy)oxane, a skeletal structure of a disaccharide, to fit in the fibroblast growth factor-1 (FGF1, the middle column of Main Figure 2). While the original ligand forms multiple hydrogen bonds with neighboring backbone atoms and the polar side chain of ASN9 (Main Figure 2(**c-2**)), DeepICL successfully designed a ligand of an identical interaction pattern with the original complex by generating phosphate and carboxylate groups instead of a sulfate (Main Figure 2(**d-2**)). Interestingly, the generated ligand formed a hydrogen bond with LYS119 via its equatorial hydroxyl group, while the original ligand possesses an axial methoxy group that is directed away from LYS119.

For the third case, we generated ligands from the benzene inside the pocket of the dihydrofolate reductase (DHFR, the right column of Main Figure 2). The original complex shows a complicated interaction pattern, including multiple salt bridges and π-π stackings (Main Figure 2(**c-3**)). The designed ligand contains a guanidine moiety similar to the original ligand to form a salt bridge and hydrogen bonds with surrounding amino acids simultaneously (Main Figure 2(**d-3**)). Additionally, the pyruvate-like group of the sampled ligand could form both a hydrogen bond and a salt bridge with ARG31. However, the sampled ligand could not interact with HIS28, which forms a salt bridge with the original ligand. The interaction similarity distributions show multi-modal distributions apparently, which happened from the construction of particular motifs that can form multiple interactions existing in the original complex. In this case, whether the model constructs a hetero-aromatic ring at the position near PHE30 and ASP26 of DHFR results in the subsequent bimodal distribution in Main Figure 2(**e-3**).

In addition, we provide five more examples of interaction-aware conditioned ligand elaboration in Supplementary Figure 1., which are composed of human sialidase (PDB ID: 2f0z), beta-lactamase Mox-1 (PDB ID: 4wbg), ubiquitin ligase (PDB ID: 6do4), abscisic acid receptor (PDB ID: 6nwc), and MAP kinase-activated protein kinase 2(PDB ID: 3fpm). With DeepICL, we sampled 100 molecules starting from the core structures of the original ligands by using the interaction condition extracted from the original complexes.

Notably, similarity distributions have dramatically diverged in the case of human sialidase (PDB ID: 2f0z). However, one might be concerned about the chemical diversity of the generated ligands since the structure of the top-1 ligand is so similar to the original ligand, placed on the first row of Supplementary Figure 1. Thus, we further provide more molecular structures of ligands that were generated to target the human sialidase in Supplementary Figure 2. While sharing the common core structure, which is oxane, generated ligands are highly diverse.

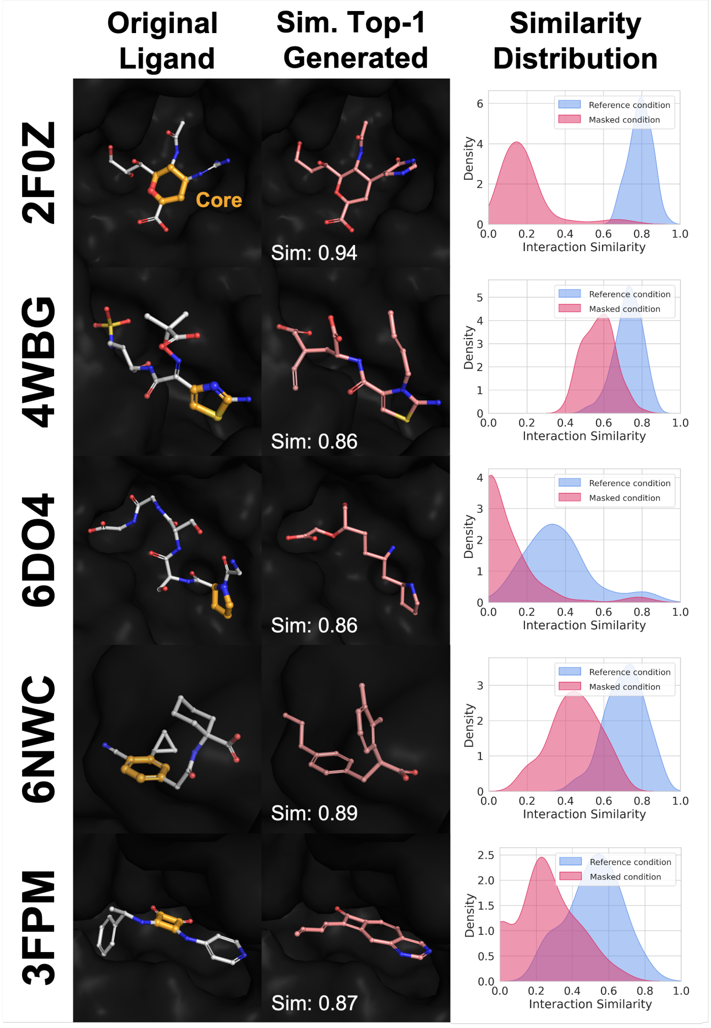

Supplementary Figure 1. Five more examples of an interaction-aware conditioned ligand elaboration task: human sialidase (PDB ID: 2f0z), beta-lactamase Mox-1 (PDB ID: 4wbg), ubiquitin ligase (PDB ID: 6do4), abscisic acid receptor (PDB ID: 6nwc), and MAP kinase-activated protein kinase 2 (PDB ID: 3fpm). Left column: structures of original ligands (white) and their core structures (orange), where the surface of surrounding pockets are shown in grey. Middle column: structures of the most similar ligand molecules elaborated from each core structure in terms of interaction similarity, and their values are denoted in the bottom left corner. Right column: distributions of interaction similarities of generated ligands, where the set of ligands that employs interaction condition clearly shows higher similarities in every case. We visualized the protein surfaces and ligand structures in 3D via PyMOL software[15].

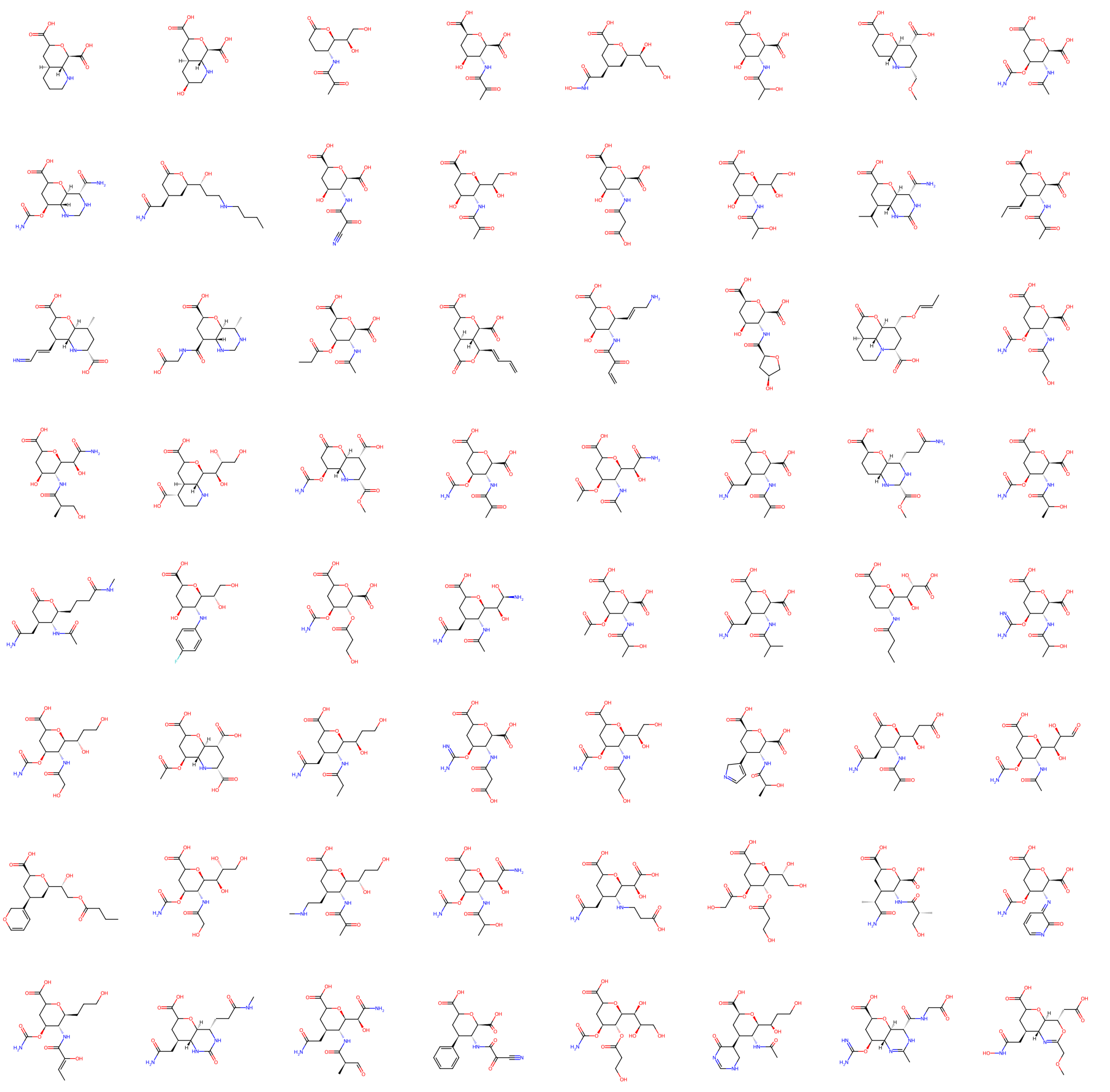

Supplementary Figure 2. Additional 64 ligands elaborated from the core structure of the original ligand binding to human sialidase (PDB ID: 2f0z) are shown. The structures are diverse despite their high interaction similarities.

Frequencies (%)	Carbon	Nitrogen	Oxygen	Fluorine
With information	63.3	14.5	21.8	0.0418
Without information	72.7	6.30	19.8	0.0205

Supplementary Table 4. The frequencies of ligand atom types that were generated within 4Å of hydrogen bond donor or acceptor protein atoms.

7. Atom addition frequency analysis

In section 2.3.2 of the main text, we discussed the number of interactions of each type and noted the number of hydrogen bonds was much lower in the case of using the model trained without interaction information. We here further provide empirical reasoning for the result by showing how the model trained with interaction information achieved more generation of hydrogen bonds. We analyzed the ligand atoms generated within 4 Å of protein atoms, which were typed as hydrogen bond acceptors or donors.

We measured the frequencies of carbon, nitrogen, oxygen, and fluorine atoms. The result is summarized in Supplementary Table 4., where the generated complexes with and without interaction information were analyzed. When the interaction information is used, the frequency of carbon is decreased, while the frequencies of nitrogen, oxygen, and fluorine are increased, facilitating the formation of more hydrogen bonds.

8. Additional discussions on geometry analysis

Supplementary Figure 3.(a) shows a distance distribution of salt bridges measured between two charge centers. The generated distribution shows a decaying pattern similar to that of the reference data. Still, our model has room for improvement as the sharp peak near 3.5 Å does not appear on the generated distribution. We speculate the reason is that a charged state of each atom is not informed to the model during generation since the protonation states can only be determined after the generation. Thus, it would be challenging to learn the exact characteristics of the salt bridge.

Supplementary Figure 3.(b) demonstrates a distribution of π-π stacking distances, which is defined between two centers of aromatic rings. The distance distribution of π-π stackings exhibits bimodal behavior, each from the parallel and the perpendicular stackings. Generating a π-π stacking geometry poses a particular challenge for our model, as it requires adding multiple atoms in the same plane to construct an aromatic ring. Moreover, the lack of explicit information about an aromatic ring center during the generation process increases the difficulty of the task; these combinatorial requirements must be fulfilled to form a π-π stacking. Although the generated distribution deviates slightly from the observed one, it apparently exhibits two distinct modes.

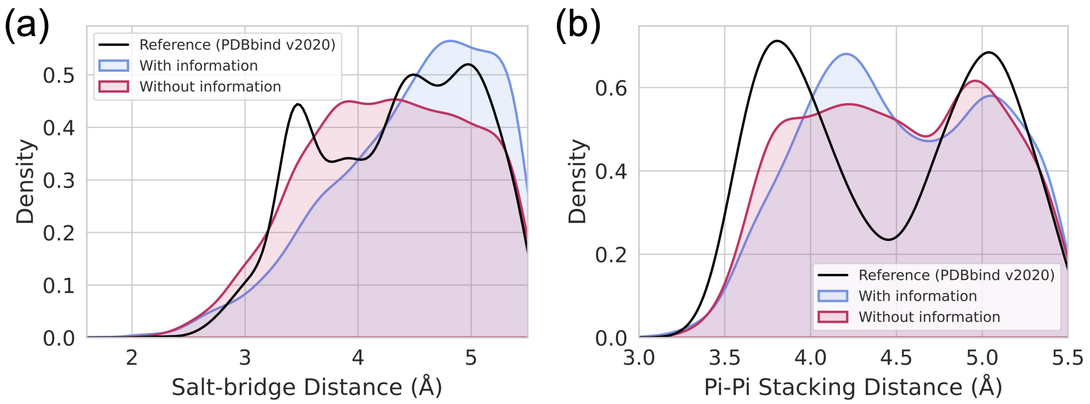

Supplementary Figure 3. Distributions of (a) salt bridge and (b) π-π stacking distances, which were measured via the PLIP software.

9. Scaffold diversity and novelty of ligands generated by DeepICL

We conducted further analysis on the ten most commonly appearing scaffolds among the generated ligands and illustrated them in Supplementary Figure 4.(a). Our model generates the benzene ring most frequently, which is also the most prevalent scaffold in the training data. Our model also generates molecules with scaffolds that are less frequently observed in the training data; diphenylmethane ranks 6th among the generated ligands, whereas it is in the 17th place in the training ligands. Tetralin shows a much larger difference: 9th in the generated ligands but 352nd in the training data. Thus, our model does not solely follow the observed structural priority of the training data. Also, a few examples of novel scaffolds in generated ligands are shown in Supplementary Figure 4.

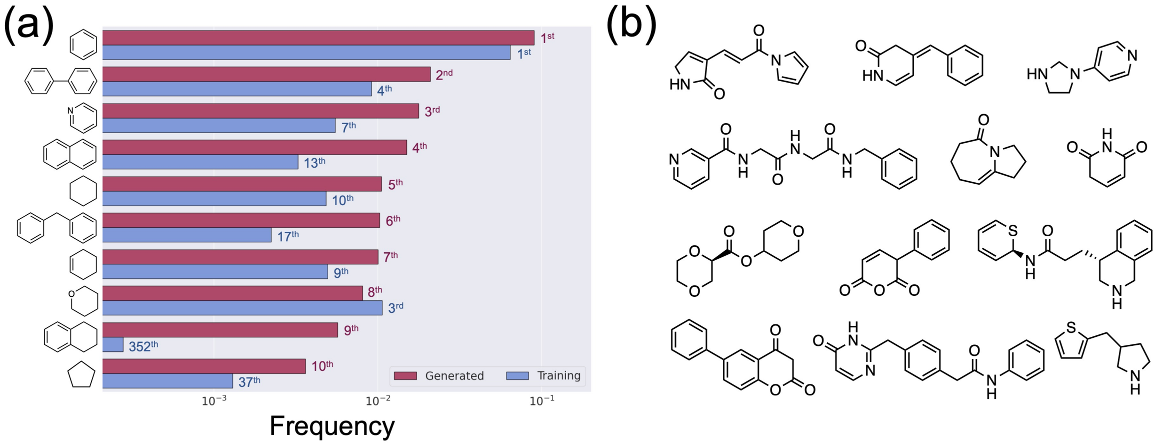

Supplementary Figure 4. (a) Bar graphs illustrating the frequency of the 10 most commonly appearing scaffolds in the generated data. For each scaffold, the graph displays the log-scaled frequencies in the generated set (red) and the training set (blue). The labeled numbers on each bar indicate rankings of their appearing frequencies in each set, respectively. (b) Few examples of novel scaffolds in generated ligands.

10. Synthesizability of ligands generated by DeepICL

We have evaluated the synthesizability of the generated molecules by using SAscore[16], a widely used metric for synthetic accessibility introduced by Etrl and Schuffenhauer. The calculated SAscores exhibited a very similar distribution to that of bioactive molecules introduced in the work of Etrl *et al.*; the average SAscore of the generated molecules was 3.18, which is close to the peak of the graph for bioactive molecules, as shown in Supplementary Figure 5. This result suggests that in terms of SAscore, the synthetic accessibility of the generated molecules is comparable to that of typical bioactive molecules.

11. Designing hinge binders of Rho-associated protein kinase 1 (ROCK1)

The ATP binding site of ROCK1 contains a hinge region that recognizes the adenine moiety through multiple hydrogen bonds[17]. Hence, ROCK1 inhibitors are often designed to bind to the hinge region, thus called hinge binders[18, 19]. We retrieved the ROCK1 structure reported by Li *et al.* (PDB ID: 3v8s)[18]. In the complex, the ligand forms hydrogen bonds with the carbonyl oxygen of GLU154 and the amide nitrogen of MET156 within the backbone of the hinge region. To design hinge binders that can form hydrogen bonds with both residues, we conditioned on two protein atoms - the carbonyl oxygen and the amide nitrogen - as hydrogen bond acceptor and hydrogen bond donor, respectively.

We generated 1,000 ligands and performed local optimization and binding affinity scoring, similar to Section 2.5 of the main text. Among the generated ligands, molecules interacting with both regions were identified as hinge binders, resulting in a total of 263 unique ligands. The binding affinity scores are shown as a jitter plot in Supplementary Figure 6.(a), where the hinge binders are colored red while other molecules

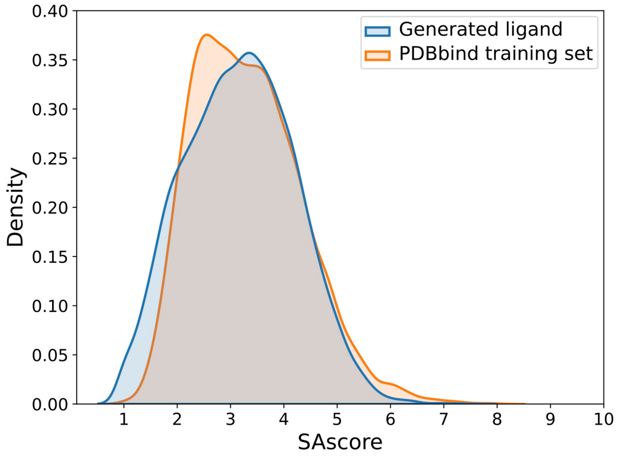

Supplementary Figure 5. Distributions of SAscores of ligands generated by DeepICL and of the training set.

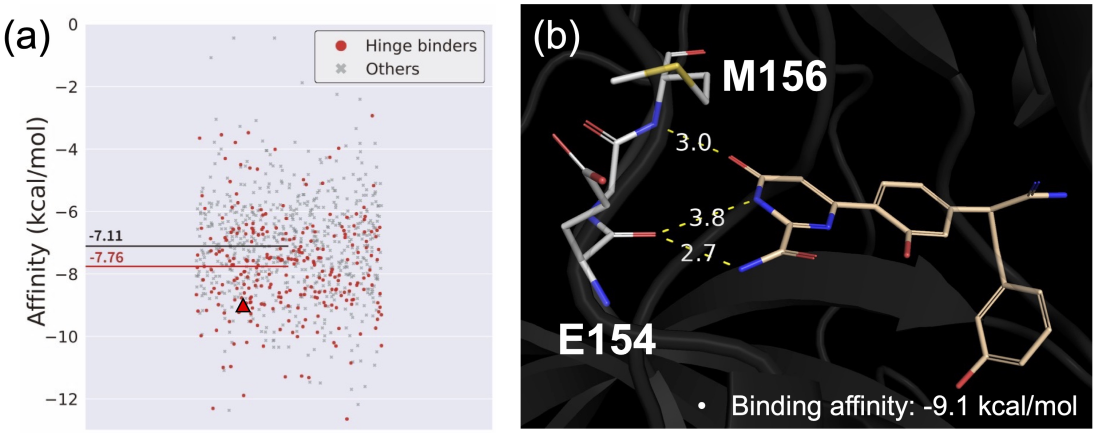

Supplementary Figure 6. (a) The jitter plot shows the binding affinity scores of the designed ligands on ROCK1; among them, hinge binders are colored red. The mean binding affinities of each group are denoted. (b) An example of a designed hinge binder inside a ROCK1 (depicted as a triangle in (a)), where the hydrogen bonds with a hinge region are depicted with yellow dashed lines. Their distances (unit: Å) are also shown.

are colored grey. Hinge-binding ligands achieved a mean binding affinity score of -7.76 kcal/mol, which was lower than that of other molecules, -7.11 kcal/mol. Consequently, the presence of interactions with the hinge region indeed contributes to the binding, emphasizing the significance of considering protein-ligand interactions in ligand design. We selected a well-designed hinge-binding ligand, depicted as a triangle in Supplementary Figure 6.(a), and visualized it in Supplementary Figure 6.(b). The molecule forms multiple hydrogen bonds with GLU154 and MET156 with plausible bond distances, achieving a fair binding affinity score of -9.1 kcal/mol.

12. Test set PDB IDs

We here provide the PDB IDs of 100 protein-ligand complexes involved in the test set.

1a4r, 1a7c, 1b56, 1dis, 1ggd, 1h60, 1hkm, 1ilh, 1lyb, 1mmr, 1njf, 1ppw, 1rdn, 1xjd, 2ael, 2drc, 2f0z, 2fqx, 2jxr, 2nnd, 2qw1, 2rc9, 2uzj, 2w68, 2xow, 2y6o, 3bm8, 3fpm, 3fxb, 3g3n, 3gsm, 3ip9, 3iqh, 3k16, 3meu, 3mj5, 3oy8, 3p3g, 3qvv, 3ta1, 3ud9, 3v2n, 3vw1, 4dfu, 4eu0, 4gki, 4i6f, 4j78, 4jj7, 4jpx, 4k42, 4k7o, 4l02, 4lar, 4mrf, 4o43, 4ocq, 4r74, 4tvj, 4tx6, 4u0n, 4wbg, 5bve, 5ii2, 5jnn, 5jz9, 5l9i, 5ldk, 5m4k, 5m77, 5mf6, 5myv, 5owc, 5uoy, 5v5e, 5wii, 5y24, 5ypy, 5z95, 5zun, 6a94, 6bto, 6chh, 6cwn, 6dik, 6do4, 6fv1, 6gvz, 6h1i, 6h4a, 6hh3, 6hly, 6hry, 6i0k, 6i68, 6iou, 6nwc, 6p10, 6qat, 6st0

Supplementary References

[1] Stefan Elfwing, Eiji Uchibe, and Kenji Doya. “Sigmoid-weighted linear units for neural network function approximation in reinforcement learning”. In: *Neural networks* 107 (2018), pp. 3–11.

[2] Junyoung Chung et al. “Empirical Evaluation of Gated Recurrent Neural Networks on Sequence Modeling”. In: *Preprint at arXiv:1412.3555* (2014).

[3] Samuel R Bowman et al. “Generating sentences from a continuous space”. In: *arXiv preprint arXiv:1511.06349* (2015).

[4] Casper Kaae Sønderby et al. “Ladder variational autoencoders”. In: *Advances in neural information processing systems* 29 (2016).

[5] Yoon Kim et al. “Semi-amortized variational autoencoders”. In: *International Conference on Machine Learning*. PMLR. 2018, pp. 2678–2687.

[6] Hao Fu et al. “Cyclical annealing schedule: A simple approach to mitigating kl vanishing”. In: *arXiv preprint arXiv:1903.10145* (2019).

[7] Adam Paszke et al. “Automatic differentiation in pytorch”. In: (2017).

[8] Niklas Gebauer, Michael Gastegger, and Kristof Schütt. “Symmetry-adapted generation of 3d point sets for the targeted discovery of molecules”. In: *Advances in neural information processing systems* 32 (2019).

[9] Junmei Wang et al. “Development and testing of a general amber force field”. In: *Journal of computational chemistry* 25.9 (2004), pp. 1157–1174.

[10] Peter Eastman et al. “OpenMM 7: Rapid development of high performance algorithms for molecular dynamics”. In: *PLoS computational biology* 13.7 (2017), e1005659.

[11] William L Jorgensen et al. “Comparison of simple potential functions for simulating liquid water”. In: *The Journal of chemical physics* 79.2 (1983), pp. 926–935.

[12] Michael P Allen and Dominic J Tildesley. *Computer simulation of liquids*. Oxford university press, 2017.

[13] James A Maier et al. “ff14SB: improving the accuracy of protein side chain and backbone parameters from ff99SB”. In: *Journal of chemical theory and computation* 11.8 (2015), pp. 3696–3713.

[14] Robert T McGibbon et al. “MDTraj: a modern open library for the analysis of molecular dynamics trajectories”. In: *Biophysical journal* 109.8 (2015), pp. 1528–1532.

[15] Warren L DeLano et al. “Pymol: An open-source molecular graphics tool”. In: *CCP4 Newsl. Protein Crystallogr* 40.1 (2002), pp. 82–92.

[16] Peter Ertl and Ansgar Schuffenhauer. “Estimation of synthetic accessibility score of drug-like molecules based on molecular complexity and fragment contributions”. In: *Journal of cheminformatics* 1 (2009), pp. 1–11.

[17] Robert Urich et al. “De novo design of protein kinase inhibitors by in silico identification of hinge region-binding fragments”. In: *ACS chemical biology* 8.5 (2013), pp. 1044–1052.

[18] Rongshi Li et al. “Fragment-based and structure-guided discovery and optimization of Rho kinase inhibitors”. In: *Journal of medicinal chemistry* 55.5 (2012), pp. 2474–2478.

[19] Paul Beroza et al. “Chemical space docking enables large-scale structure-based virtual screening to discover ROCK1 kinase inhibitors”. In: *Nature Communications* 13.1 (2022), p. 6447.
